# Profiling the *Plasmodium falciparum* Erythrocyte Membrane Protein 1–Specific Immununoglobulin G Response Among Ghanaian Children With Hemoglobin S and C

**DOI:** 10.1093/infdis/jiad438

**Published:** 2023-10-06

**Authors:** Andrew V Oleinikov, Zakaria Seidu, Irina V Oleinikov, Mary Tetteh, Helena Lamptey, Michael F Ofori, Lars Hviid, Mary Lopez-Perez

**Affiliations:** Charles E Schmidt College of Medicine, Florida Atlantic University, Boca Raton, Florida, USA; Centre for Medical Parasitology, Department of Immunology and Microbiology, Faculty of Health and Medical Sciences, University of Copenhagen, Copenhagen, Denmark; Department of Immunology, Noguchi Memorial Institute for Medical Research, College of Health Sciences, University of Ghana, Accra, Ghana; West Africa Centre for Cell Biology of Infectious Pathogens, Department of Biochemistry, Cell and Molecular Biology, University of Ghana, Accra, Ghana; Charles E Schmidt College of Medicine, Florida Atlantic University, Boca Raton, Florida, USA; Department of Medical Diagnostics, Faculty of Allied Health Sciences, Kwame Nkrumah University of Science and Technology, Kumasi, Ghana; Department of Immunology, Noguchi Memorial Institute for Medical Research, College of Health Sciences, University of Ghana, Accra, Ghana; Department of Immunology, Noguchi Memorial Institute for Medical Research, College of Health Sciences, University of Ghana, Accra, Ghana; Centre for Medical Parasitology, Department of Immunology and Microbiology, Faculty of Health and Medical Sciences, University of Copenhagen, Copenhagen, Denmark; Centre for Medical Parasitology, Department of Infectious Diseases, Rigshospitalet, Copenhagen, Denmark; Centre for Medical Parasitology, Department of Immunology and Microbiology, Faculty of Health and Medical Sciences, University of Copenhagen, Copenhagen, Denmark

**Keywords:** Ghana, malaria, PfEMP1, PfHRP2, sickle cell trait

## Abstract

Members of the *Plasmodium falciparum* erythrocyte membrane protein 1 (PfEMP1) family are important targets for protective immunity. Abnormal display of PfEMP1 on the surfaces of infected erythrocytes (IEs) and reduced cytoadhesion have been demonstrated in hemoglobin (Hb) AS and HbAC, inherited blood disorders associated with protection against severe *P. falciparum* malaria. We found that Ghanaian children with HbAS had lower levels of immunoglobulin G against several PfEMP1 variants and that this reactivity increased more slowly with age than in their HbAA counterparts. Moreover, children with HbAS have lower total parasite biomass than those with HbAA at comparable peripheral parasitemias, suggesting impaired cytoadhesion of HbAS IEs in vivo and likely explaining the slower acquisition of PfEMP1-specific immunoglobulin G in this group. In contrast, the function of acquired antibodies was comparable among Hb groups and appears to be intact and sufficient to control parasitemia via opsonization and phagocytosis of IEs.

## Teaser

Heterozygous carriers of sickle hemoglobin (HbAS) had reduced total and sequestered parasite biomass and lower immunoglobulin G response to *Plasmodium falciparum* erythrocyte membrane protein 1 variants associated with severe malaria. Moreover, reactivity increased more slowly with age in those individuals.

*Plasmodium falciparum* causes most malaria cases and almost all the associated mortality and severe morbidity. Hence, it is not surprising that this parasite has exerted a strong evolutionary pressure on the human genome, selecting polymorphisms that protect against severe malaria [1]. These include structural variants of adult hemoglobin (HbA; wild type) involving a single point mutation within codon 6 of the β-globin as in hemoglobin (Hb) S and C. Heterozygous carriers (HbAS or HbAC) are highly protected against severe malaria but not from *P. falciparum* infection [1], suggesting a multifactorial protection.

Protective immunity to malaria is mainly antibody mediated, and members of the *P. falciparum* erythrocyte membrane protein 1 (PfEMP1) family expressed on the surface of the infected erythrocytes (IEs) are important targets [2, 3]. To evade the immune response, the parasites can switch transcription among the approximately 60 different *var* genes encoding PfEMP1 and express a single variant at a time [4]. The *var* genes can be divided into groups based on their genomic location and structural features [5]. The different groups contain specific Duffy binding–like (DBL; α, β, γ, δ, ε, and ζ) domains and cysteine-rich interdomain regions (α, β, and γ) [6] that mediate the binding to host receptors such as CD36, intercellular adhesion molecule 1, or EPCR [5]. Thus, group A or B/A proteins are particularly associated with severe malaria, whereas the more diverse group B and C are commonly found among uncomplicated and asymptomatic infections [5].

IE adhesion to specific endothelial host receptors (ie, cytoadhesion/sequestration) or to surrounding uninfected erythrocytes (ie, rosetting) [7] allows IEs to evade destruction in the spleen and lead to tissue inflammation. Reduced display of PfEMP1 on the IE surface [8–11] and decreased cytoadhesion [9, 12] and rosetting [11] have been demonstrated in HbAS and HbAC, which perhaps limit in vivo sequestration and therefore reduce the risk of severe malaria. A higher frequency of memory CD8^+^ T cell [13] and a novel subset of memory-activated natural killer cells [14] in HbAS have been proposed to contribute to parasite density control.

The degree of reduction in malaria infection and parasite density in HbAS individuals seems to increase with age [15], suggesting that acquired immunity to malaria in those individuals also plays an important role. However, mixed results have been reported regarding differences in the antibody response to several malaria antigens [16–19] and PfEMP1-specific antibodies have received very limited attention [19–21]. Thus, on the assumption that HbAS and HbAC protect predominantly against PfEMP1 variants associated with severe malaria, we measured plasma levels of PfEMP1- and non–PfEMP1-specific immunoglobulin (Ig) G in Ghanaian children, the ability of specific IgG to inhibit rosetting and to opsonize IEs for phagocytosis, and the total parasite biomass, which includes both the circulating and the sequestered IEs.

## METHODS

### Ethical Statement

The study was approved by the Ethics Review Committee of the Ghana Health Service (GHS-ERC 008/07/19), the Noguchi Memorial Institute for Medical Research Institutional Review Board (CPN 006/19), and Kwame Nkrumah University of Science and Technology (CHRPE/AP/308/19). Declarations of free willingness to participate in the study and written informed consent were obtained from all participants or guardians before enrollment.

### Study Site and Participants

We used plasma samples collected during 2 independent cross-sectional studies conducted in Ghana, where malaria transmission is generally perennial. The first study [22] was a community-based study conducted during the rainy season in rural communities in Northern Ghana. Children from randomly selected households were recruited for sample collection and *P. falciparum* screening. The second study [23] enrolled children with uncomplicated malaria attending the outpatient department of the Begoro District Hospital in Eastern Ghana, a region of intense malaria transmission. Hb phenotypes and *P. falciparum* infections were determined by means of isoelectric focusing electrophoresis and polymerase chain reaction (PCR), respectively [23, 24]. Hb phenotypes such as HbF (1.2%), homozygous (6%), and heterozygous (30%) α-thalassemia are also found in the study region (Z. S., unpublished data).

A subset of plasma samples collected in both study sites from children with *P. falciparum* infection confirmed by PCR was used to measure the concentration of *P. falciparum* histidine-rich protein 2 with the Quantimal CELISA kit (Cellabs), following the manufacturer's instructions. Parasite biomass (total, circulating, and sequestered) was calculated as described elsewhere [25]. We used a second subset of 120 plasma samples collected in Northern Ghana from children with HbAA, HbAS, and HbAC and matched 1:1:1 by age to analyze specific IgG response. All samples were processed in a blinded fashion for the Hb phenotype.

### Recombinant Proteins

Full-length PfEMP1 (HB3VAR06, IT4VAR09, and IT4VAR60) recombinant proteins were produced in baculovirus-transfected Sf9 insect cells [26]. Two DBLβ domains (HB3VAR34 and PFD1235w) [27], the domain R0 of glutamate-rich protein (GLURP) [28], and a nontoxic tetanus toxin C-fragment were produced in *Escherichia coli*. *P. falciparum* merozoite surface protein 3 (PfMSP3) [29] and *P. falciparum* 230-kDa sexual-stage protein (Pfs230) [30] were produced in *Lactococcus lactis*, whereas *P. falciparum* circumsporozoite protein (PfCSP) was synthesized by Genscript. A crude lysate of asexual stages was prepared from *P. falciparum* 3D7. In addition, 46 single- or double-PfEMP1 domain proteins from *P. falciparum* 3D7 expressed in COS7 cells, as described elsewhere [31] and covering the same number of different *var* genes, were used.

### Specific IgG Response to Recombinant Proteins by Enzyme-Linked Immunosorbent Assay

IgG reactivity against recombinant proteins was measured by means of enzyme-linked immunosorbent assay (ELISA) as described elsewhere [20]. Briefly, plasma samples (1:400) followed by horseradish peroxidase–conjugated rabbit anti-human IgG (1:3000; Dako) were added to 96-well flat-bottom microtiter plates (Nunc MaxiSorp; Thermo Fisher Scientific) previously coated with recombinant proteins. Bound antibodies were detected by adding TMB PLUS2 (Eco-Tek), and the reaction stopped with 0.2 mol/L sulfuric acid. The optical density was read at 450 nm (HiPo MPP-96 microplate reader; Molecular Devices), and the specific antibody levels were calculated in arbitrary units, as described elsewhere [20]. Plasma samples from Danish adults without malaria exposure and a pool of Ghanaian adults with previous *P. falciparum* infection were included as negative and positive controls, respectively. Negative cutoff values were calculated as the mean arbitrary unit values plus 2 standard deviations obtained with the negative control samples.

### Multiplex Immunoassays for PfEMP1 Proteins

IgG reactivity to recombinant PfEMP1 proteins from *P. falciparum* 3D7 was assessed by means of a multiplex immunoassay using 46 single- or double-domain proteins (Supplementary Table 1) immobilized on BioPlex beads, as described elsewhere [31, 32]. Briefly, plasma samples preabsorbed with IgG from goat serum were diluted (1:200) and mixed with coated beads (10–13 bead regions), followed by detection with phycoerythrin–goat anti-human IgG (1:250). IgG binding to bead-bound constructs was measured in duplicates on a BioPlex 200 machine (BioRad). Beads with an immobilized HisAdEx construct [31] were used as a negative control. This construct contained all the same parts as recombinant domain constructs but short irrelevant 37-mer peptides instead of PfEMP1 domains.

To account for plate-to-plate variation, median fluorescence intensities (MFIs) were normalized (nMFIs) using the mean signal of the negative control construct in each sample. Plasma samples from Danish and US adults without malaria exposure were included as negative controls to calculate the cutoff, defined as the average nMFI values plus 2 standard deviations. Recognition was defined as an nMFI higher than the cutoff in ≥1 sample. For categorical analysis, nMFIs were scored from 0 (reactivity below cutoff) to 3 (higher than the cutoff by >1000 nMFI), allowing comparison between PfEMP1 proteins (IgG reactivity scores).

### Malaria Parasite Culture and PfEMP1 Selection

*P. falciparum* clones IT4/FCR3 and HB3 were maintained in serum-free Roswell Park Memorial Institute 1640 medium, as described elsewhere [33]. Late-stage IEs of HB3 parasites were selected for surface expression of HB3VAR06 using protein A–coupled DynaBeads coated with a specific rabbit antiserum [26], as described elsewhere [34]. A similar approach was used to select IT4 IEs for expression of IT4VAR09 or IT4VAR60. IE surface expression of the corresponding PfEMP1 protein was monitored by flow cytometry, as described elsewhere [34]. A CytoFLEX S flow cytometer (Beckman Coulter Life Sciences) was used for data acquisition, and the data were analyzed with FlowLogic software (version 8.3; Inivai Technologies). MFI data were normalized to the MFI on non–malaria-exposed samples.

### Rosetting Assays

The ability of plasma samples to disrupt rosettes was tested by flow cytometry as described elsewhere [35]. Briefly, late-stage HbAA IEs at 2% hematocrit in 10% human serum Roswell Park Memorial Institute 1640 medium were incubated for 1 hour at 37°C with plasma samples (1:10). Parasite nuclei were stained with Hoechst 33342 (10 µg/mL; Invitrogen) and dihydroethidium (5 μg/mL; Invitrogen). Late-stage IEs (Hoechst and dihydroethidium positive) were gated using forward scatter (FSC-A versus FSC-H) to determine the percentage of multiplets (rosettes) and the mean side scatter (SSC-A) for rosette size. Rosetting rates and rosette sizes were calculated relative to values without specific PfEMP1 antibodies (control for maximum rosetting), as described elsewhere [35].

### Antibody-Dependent THP-1 Cell Phagocytosis Assay

High-throughput flow cytometry-based antibody-dependent cellular phagocytosis (ADCP) assays were performed using undifferentiated THP-1 cells, as described elsewhere [36]. Nuclei of purified late-stage IEs were stained with ethidium bromide (10 μg/mL). For opsonization, stained IEs were incubated for 30 minutes at 37°C with plasma samples (1:10), followed by coincubation with THP-1 cells (IE/THP-1 ratio, 10:1) for 40 minutes at 37°C in a 5% CO_2_ atmosphere to allow phagocytosis. Nonphagocytized IEs were removed using ammonium chloride lysing solution. Data acquisition and analysis were performed as described above. The percentage of THP-1 cells that had phagocytosed IEs (THP-1 cells positive for ethidium bromide) was calculated relative to the positive control (control for maximum phagocytosis).

### Statistical Analysis

Data were analyzed and plotted using GraphPad Prism software, version 9.5 (GraphPad Software). The sample sizes and specific statistical tests are indicated in the text or in the figures. Differences were considered statistically significant at *P* < .05. For the ELISA and multiplex assays, the breadth of the IgG reactivity for each group of antigens was defined as the number of recognized antigens divided by the number of tested antigens in the group multiplied by the number of plasma samples tested with each group of antigens. Seroprevalence was defined as the proportion of samples recognizing an individual antigen or group of antigens.

## RESULTS

### Reduced Total Parasite Biomass in Children With HbAS

**Figure 1.**
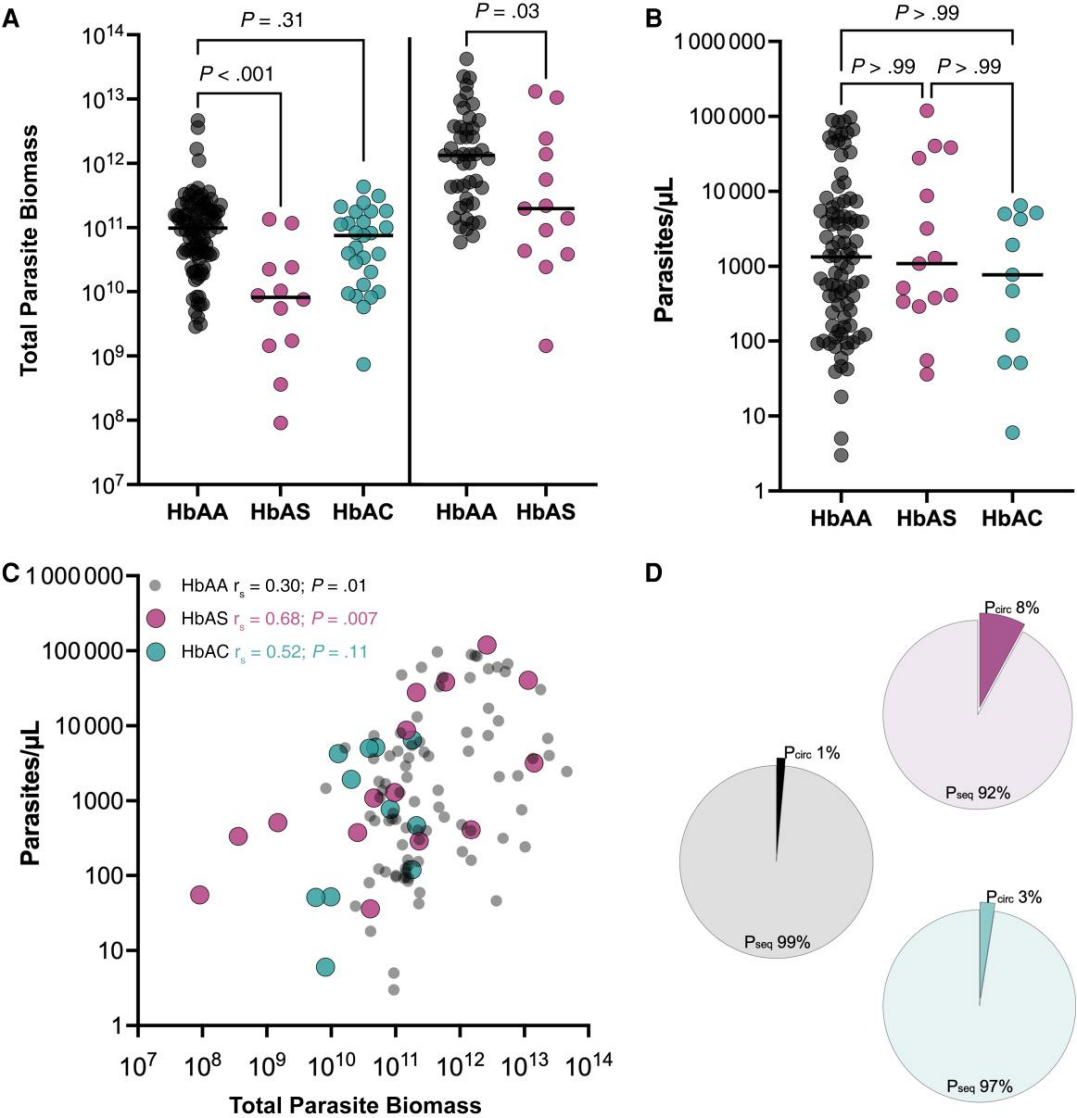
Lower parasite biomass in children with hemoglobin (Hb) AS. *A,* Total parasite biomass in 135 children with *Plasmodium falciparum* infection (*left*) and 58 with uncomplicated *P. falciparum* malaria (*right*). *B*, Microscopic parasitemia (in parasites per microliter) in 116 children from both cohorts. Horizontal lines indicate median values. *P* values were calculated using Kruskal-Wallis test followed by Dunn multiple comparison or Mann-Whitney test. *C*, Spearman rank correlation between parasitemia and total parasite biomass in all children (n = 116). *D*, Sequestered (P_seq_) and circulating (P_circ_) parasite biomasses were calculated using plasma *P. falciparum* histidine-rich protein 2 concentrations. Data were averaged and used to create the pie charts.

Using an availability-based subset of 135 plasma samples from children with confirmed *P. falciparum* infection [22] (Supplementary Table 2), we observed that those with HbAS had significantly lower plasma concentrations of *P. falciparum* histidine-rich protein 2 concentrations (*P* < .001), corresponding to approximately 8 times lower total parasite biomass than in HbAA (Figure 1*A*). In contrast, the parasite biomass in children with HbAC did not differ significantly from that in those with HbAA. Those findings were validated using a subset of 58 samples from children with uncomplicated *P. falciparum* malaria [23] (Supplementary Table 3). We found that sick children with HbAS had approximately 7 times smaller total parasite biomass than corresponding children with HbAA (Figure 1*A*). Overall, microscopic parasitemias were comparable among groups (Figure 1*B*); however, a strong positive correlation with total parasite biomass was observed only in HbAS (*r_s_* = 0.68; *P* = .007) (Figure 1*C*), suggesting a lower sequestered mass in HbAS. Indeed, the average proportion of sequestered biomass was higher in HbAA (99%) than in HbAS (92%) and HbAC (97%; *P* = .13) (Figure 1*D*).

### IgG Responses to Non-PfEMP1 Antigens

**Figure 2.**
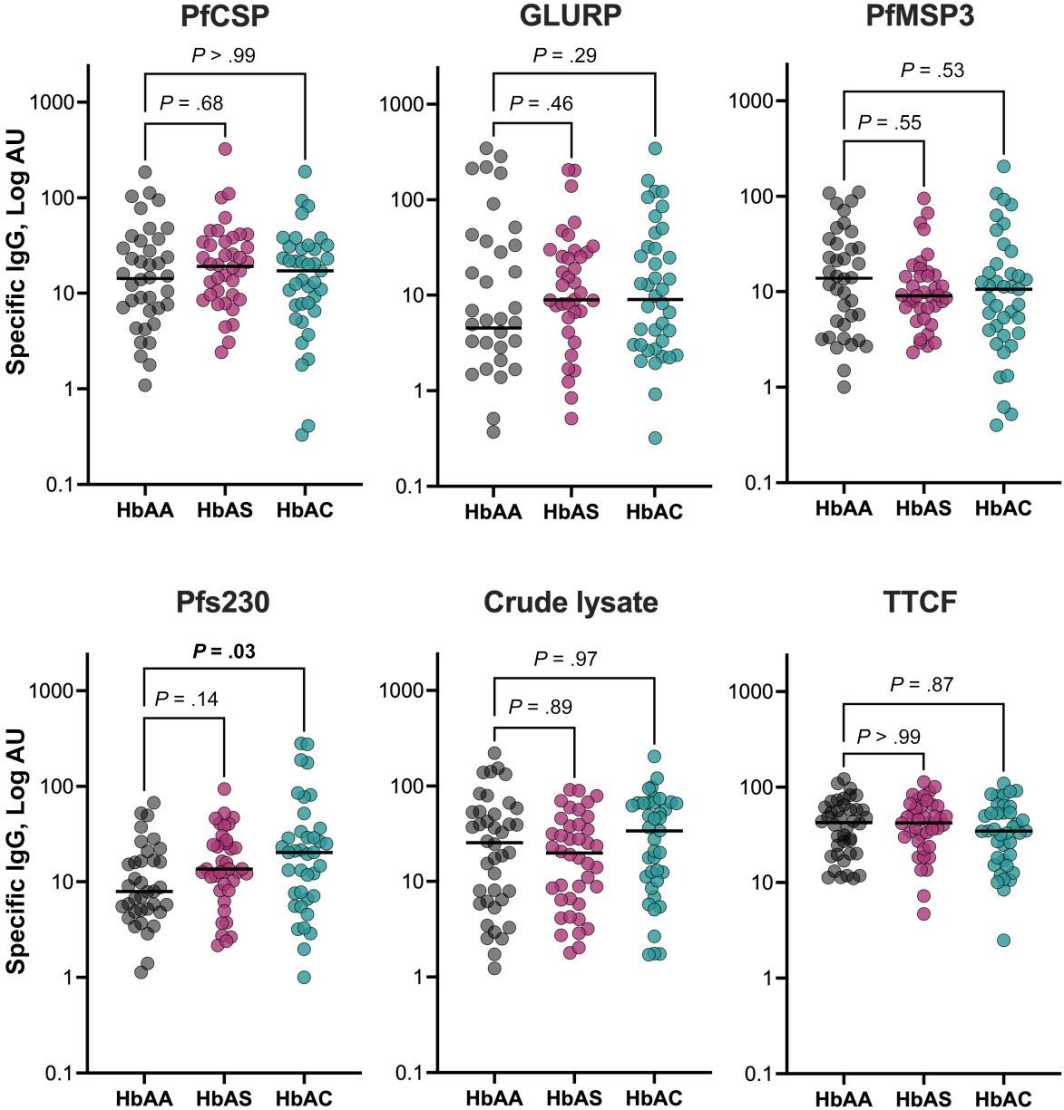
Antibody response to malaria antigens. Specific immunoglobulin (Ig) G levels against malaria antigens from several parasite stages, sporozoites (*P. falciparum* circumsporozoite protein [PfCSP], n = 119), merozoites (glutamate-rich protein [GLURP], *P. falciparum* merozoite surface protein 3 [PfMSP3]), n = 119), gametocytes (*P. falciparum* 230-kDa sexual-stage protein [Pfs230], n = 118), crude lysate of asexual blood stages (n = 119), and a nonmalaria antigen (tetanus toxin C-fragment [TTCF], nontoxic TTCF, n = 120) were determined by an enzyme-linked immunosorbent assay in plasma samples from 120 children with hemoglobin (Hb) AA, HbAS, and HbAC. Horizontal lines indicate median values. *P* values were calculated using Kruskal-Wallis test followed by Dunn multiple comparison test. All panels express the values in log arbitrary units (AU).

We next explored the specific IgG response to malaria antigens using a second subset of plasma samples collected in Northern Ghana from children with HbAA (n = 40), HbAS (n = 40), or HbAC (n = 40) matched by age (median age [IQR], 6 [4–9] years; Table 1). Although some differences were observed among Hb genotypes (Figure 2), only levels of IgG to Pfs230 were significantly higher in HbAC than in HbAA (20.2 vs 7.9, respectively; *P* = .03), particularly in those PCR positive (30.7 vs 9.8; *P* = .02). Age was positively correlated with antibody responses to all malaria antigens (*r_s_* = 0.20–0.30; *P* < .05). In contrast, levels of antibody to tetanus toxin C-fragment (TTCF) were less variable and similar among the groups.

**Table 1.** Clinical parameters by hemoglobin phenotype

Parameter	Children, No. (%)^a^				*P* Value^b^
	HbAA (n = 40)	HbAS (n = 40)	HbAC (n = 40)	Total (n = 120)	
Female sex	16 (40)	12 (30)	14 (35)	42 (35)	.64
PCR positive	5 (13)	9 (23)	13 (33)	27 (23)	.10
Submicroscopic parasitemia^c^	3 (8)	5 (13)	6 (15)	14 (12)	.57
Hb, median (IQR) g/dL	10.9 (10.3–11.9)	11.0 (10.0–11.6)	10.8 (10.1–11.3)	10.9 (10.2–11.6)	.60

Abbreviations: Hb, hemoglobin; IQR, interquartile range; PCR, polymerase chain reaction.

^a^Data represent no. (%) of children unless otherwise specified.

^b^ *P* values calculated using χ^2^ test for qualitative and Kruskal-Wallis test for quantitative variables.

^c^Positive by PCR but negative by microscopy.

### PfEMP1-Specific IgG Responses

**Figure 3.**
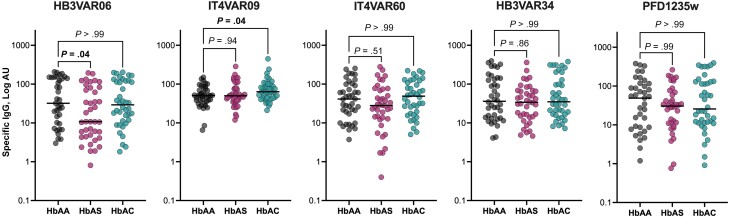
Antibody response to recombinant *Plasmodium falciparum* erythrocyte membrane protein 1 (PfEMP1) proteins. Specific immunoglobulin (Ig) G levels against a full-length recombinant (HB3VAR06, IT4VAR09, and IT4VAR60) and Duffy binding–like β (HB3VAR34 and PFD1235w) PfEMP1 proteins in hemoglobin (Hb) AA, HbAS, and HbAC were determined by an enzyme-linked immunosorbent assay (n = 120). Horizontal lines indicate median values. *P* values were calculated using Kruskal-Wallis test followed by Dunn multiple comparison test. All panels express the values in log arbitrary units (AU).

We hypothesized that the clinical protection against severe *P. falciparum* malaria afforded to HbAS and HbAC individuals involves IgG specifically targeting PfEMP1 variants associated with severe malaria in children. We therefore first evaluated levels of IgG specific for 3 PfEMP1 proteins mediating both rosetting and Fc-dependent IgM binding [37] and 2 intercellular adhesion molecule 1–binding DBLβ domains, which also bind CD36 (HB3VAR34) or EPCR (PFD1235w) [38]. Compared with those in children with HbAA, levels of HB3VAR06-specific IgG were significantly lower in children with HbAS, and levels of IT4VAR09-specific IgG were higher in HbAC (Figure 3). Corresponding differences were observed for levels of plasma IgG recognizing HB3VAR06-positive IEs (Supplementary Figure 1).

**Figure 4.**
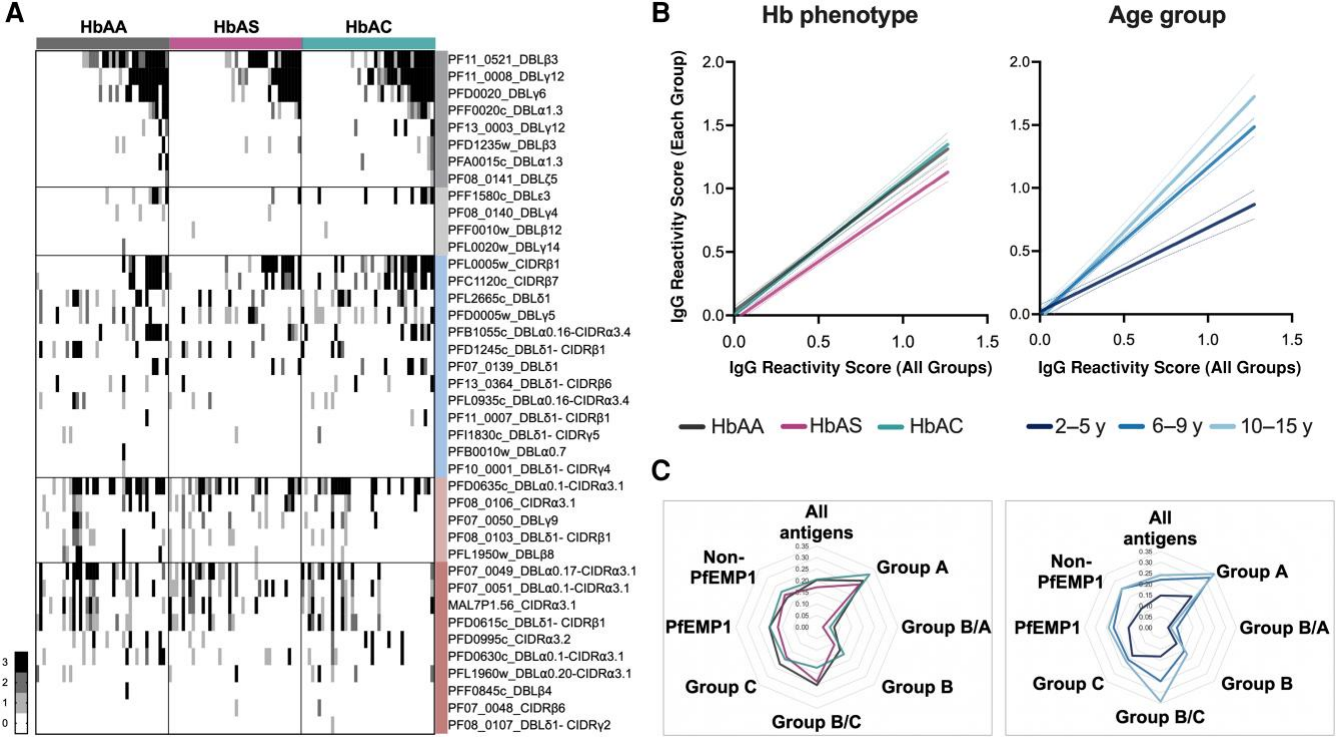
Specific immunoglobulin (Ig) G response to single and double *Plasmodium falciparum* erythrocyte membrane protein 1 (PfEMP1) domains. *A*, Heatmap showing IgG reactivity scores to each of the 40 PfEMP1 proteins in 120 children (37 hemoglobin [Hb] AS and 39 HbAC tested for groups A and B/A). Individuals were ranked according to reactivity (gray-scale bar) and grouped according to the Hb genotype. Proteins are ordered and color coded according to *var* gene group (group A, group B/A, group B, group B/C, group C). *B*, Simple linear regression and 95% confidence interval (*dotted lines*) in which the average reactivity to a particular PfEMP1 tested in the beads array with plasma samples from each Hb phenotype (HbAA, HbAS, or HbAC) or age group (2–5, 6–9, or 10–15 years) are plotted against the average of 3 groups; the slope of the regression line is proportional to the overall intensity in each group. *C*, Radar chart shows the breadth of IgG reactivity for each group of malaria antigens according to the Hb phenotype (*left*) or age group (*right*). Distance to the center point constitutes the reactivity of the respective group. All antigens (n = 50) include both PfEMP1 and non-PfEMP1 proteins.

Next, we analyzed plasma IgG levels specific for 46 PfEMP1 domains using a custom-made bead array [31]. Overall, 40 of the domains tested were recognized by IgG in the plasma from ≥1 child, and only those proteins were included in further analysis. Domain-specific IgG levels were generally lower in children with HbAS than in those with HbAA (*F* = 3.15; *P* = .04) (Figure 4*A* and 4*B*). Seroprevalence of domain-specific IgG varied from 1% to 60% (Supplementary Figure 2). However, significant differences between HbAS (13%) and HbAA (30%) or HbAC (30%) were observed only for domains from the integrin αVβ3-binding group B PfEMP1 variant PFL2665 [39] and the group C variant PFD0995c (5% for HbAS vs 20% for HbAA; *P* = .04 [χ^2^ test]). Moreover, in children with HbAA or HbAC, domains from ≥3 of 5 EPCR-binding PfEMP1 tested were recognized, whereas HbAS recognized only 2, with the highest seroprevalence for PF11_0521 (52%) (Supplementary Figure 2). Significant differences were also observed when children were stratified by age in 3 groups, with those <5 years old having the lowest reactivity to PfEMP1 domains (*F* = 33.71; *P* < .001) (Figure 4*B*).

IgG to all PfEMP1 antigens (*P* = .01; χ^2^ test) and combined group A and B/A (*P* = .05; χ^2^ test) was detected less often in children with HbAS than in those with HbAC or HbAA (Figure 4*C*), particularly in children with *P. falciparum* infection (*P* = .02). In contrast, group B domains were more frequently recognized in children with HbAC (*P* = .03; χ^2^ test). Children <5 years old had significantly lower reactivity to both PfEMP1 and non-PfEMP1 antigens (Figure 4*C*), and age was positively correlated with the breadth of response. However, when the Hb phenotype was considered, the correlation with all PfEMP1, group A, and B/A was significant only for children with HbAA (Supplementary Figure 3).

### Disruption of Rosetting by PfEMP1-Specific IgG From Children With HbAS or HbAC

**Figure 5.**
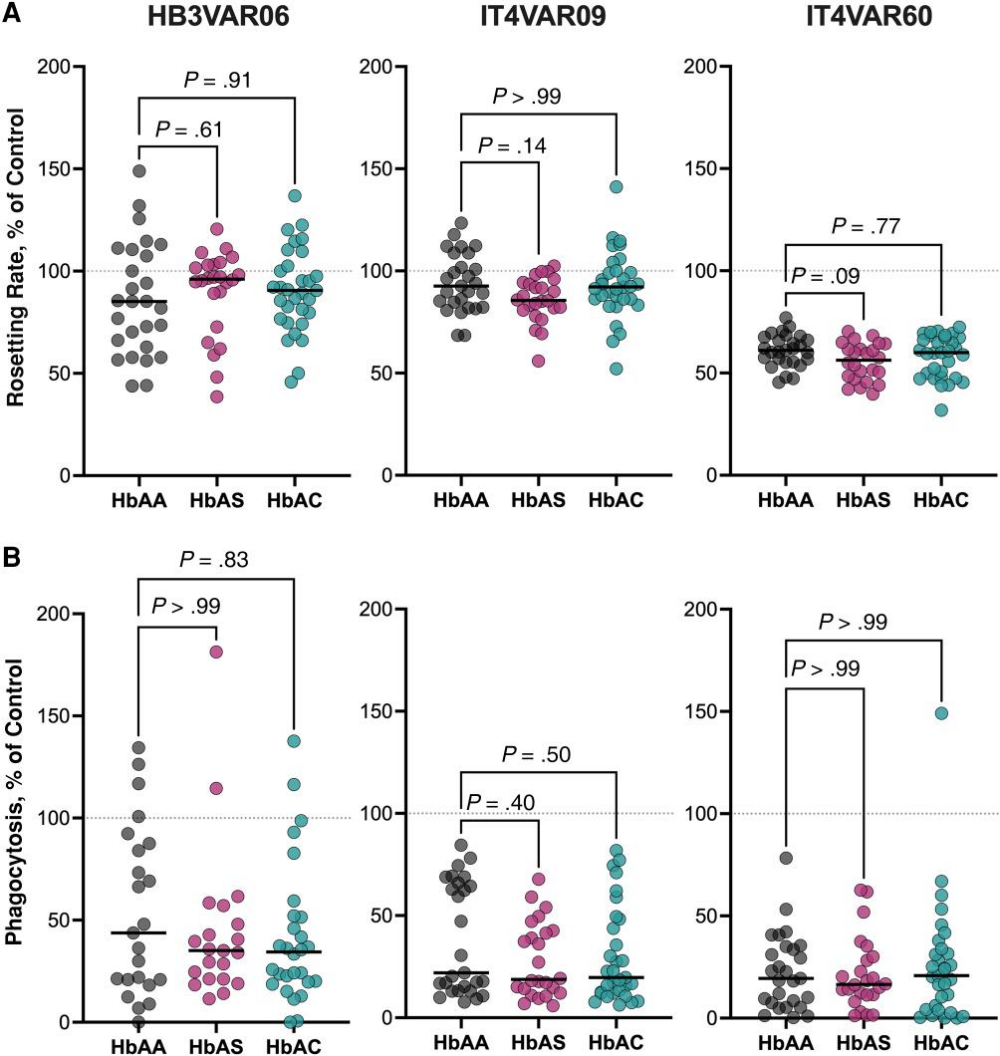
Rosette disruption and *Plasmodium falciparum* erythrocyte membrane protein 1 (PfEMP1)–specific immunoglobulin (Ig) G dependent phagocytosis of infected erythrocytes (IEs). *A*, Rosette disruption by plasma from hemoglobin (Hb) AA (n = 27), HbAS (n = 31), and HbAC (n = 24) children was measured as the rosetting rate relative to the absence of specific PfEMP1 antibodies (control for maximum resetting [*dotted line*]). *B*, Phagocytosis of IEs by plasma from HbAA (n = 28), HbAS (n = 31), and HbAC (n = 24) children relative to the positive control (control for maximum phagocytosis [*dotted line*]). Horizontal lines indicate median values. *P* values were calculated using Kruskal-Wallis test followed by Dunn multiple comparison test.

Because anti-rosetting antibodies are associated with protection from severe malaria [7], we evaluated whether PfEMP1-specific antibodies from children with HbAS and HbAC were better at disrupting rosettes. Plasma samples from most of the children were able to disrupt rosettes (*P* < .001), with no differences among Hb groups (Figure 5*A*). The rosette size was almost unaffected (Supplementary Figure 4). Plasma from older children was better at disrupting rosettes (Supplementary Figure 5).

### ADCP of *P. falciparum*–Infected Erythrocytes

Besides neutralizing the binding of IEs to host receptors, PfEMP1-specific antibodies also promote ADCP of IEs, which is lower in children with severe malaria [40, 41]. Phagocytosis of IEs opsonized by plasma IgG from the children was variable and moderate for all 3 parasite clones tested (Figure 5*B*), with no significant differences among the Hb groups. However, plasma from younger children (aged 2–5 years) was less efficient in inducing phagocytosis (Supplementary Figure 5).

## DISCUSSION

Individuals carrying a mutated allele in Hb (HbAS or HbAC) are infected by *P. falciparum* at similar rates as HbAA individuals, but their parasite densities tend to be lower [15], and the infections rarely progress to severe malaria [1]. Since the reduced expression of PfEMP1 on the IE surface and the less efficient binding to host receptors [8–12] may potentially induce a differential antibody response in HbAS or HbAC individuals, we explored this using a broad repertoire of PfEMP1 proteins. We found that the sequestered and total parasite biomasses were lower in children with HbAS than in those with HbAA, suggesting decreased cytoadhesion in vivo in the former group. Second, children with HbAS had lower IgG-specific responses to groups A and B/A PfEMP1 proteins, and the reactivity increased more slowly with age. Finally, we documented that the acquired PfEMP1-specific IgG was a mixture of neutralizing and nonneutralizing IgG in all groups, with similar anti-rosetting and opsonic capacity.

Our findings agree with previous results showing that HbAS and HbAC do not protect against *P. falciparum* infection [15]. Our observation that the total, and in particular the sequestered, parasite biomass in vivo was substantially lower in children with HbAS than in those with HbAA is consistent with earlier in vitro findings of reduced PfEMP1 expression in HbAS and HbAC IEs [8–11] and decreased IE adhesion to human microvascular endothelial cells and surrounding uninfected erythrocytes [9, 11, 12]. Nevertheless, the effect of immune cellular responses cannot be ruled out as a distinctive response has been observed between HbAS and HbAA, with an elevated response in HbAS associated with parasite density control [13, 14].

Despite the trend of high levels of IgG to non-PfEMP1 antigens in HbAS, no significant differences were observed, as others have described [16–19]. Nevertheless, using a larger sample size, our group recently observed significantly higher PfCSP-specific IgG levels in children with HbAS [24]. These findings and the higher levels of IgG to Pfs230 in both HbAS and HbAC are consistent with the longer duration of infection and higher probability of having gametocytes described recently for HbAS [42]. In contrast, the similar response to TTCF suggests that HbAS and HbAC develop early acquired immunity, as suggested before for nonmalaria antigens [17, 18].

Although it is not clear whether naturally acquired immunity to noncerebral severe malaria can be achieved after only 1 or 2 episodes [43] or requires more episodes [44], it is generally agreed that this immunity is mediated mainly by PfEMP1-specific IgG [2, 3], which can block IE adhesion to clinically significant host receptors. Previous studies among individuals living in malaria-endemic areas have shown that the acquisition of IgG antibodies against PfEMP1 variants is ordered, with antibodies against group A and B/A variants being acquired earlier in life than those specific for group B and C variants [45, 46]. The former antibody group is more likely in children with uncomplicated malaria, is associated with protection from severe malaria, and is boosted after a severe episode [47]. Consistent with that, we found that the youngest children had the lowest reactivity to PfEMP1 domains tested in this study.

Although significant differences in the seroprevalence were observed for only 2 domains, recognition of group A and B/A variants associated with severe malaria was lower in children with HbAS, and the reactivity increased more slowly with age compared with findings in HbAA. This agrees with a previous observation that antibody response to another malaria antigen, Pf155/ring-infected erythrocyte surface antigen (RESA), increases more rapidly in HbAA than in HbAS [17] as well as another study showing that the protective effect of Hb status disappears after 6 years of age, when it is obscured by general premunition [16]. The lower HB3VAR06-specific IgG levels observed here in children with HbAS compared with HbAA, which was not found in our group’s earlier study in adults [20], are consistent with that. These data suggest that the reduction of PfEMP1 reactivity reflects lower levels of these antigens during previous malaria episodes in children with this phenotype.

PfEMP1-specific antibodies can block cytoadhesion and rosetting (ie, neutralizing antibodies) and opsonize IEs for clearance via ADCP [40, 41]. Higher rosette disruption with plasma from older children is consistent with the early-in-life acquisition of antibodies targeting PfEMP1 variants responsible for rosetting, thus facilitating the clearance of IEs and perhaps contributing to protection against severe malaria, as has been reported elsewhere [7, 48]. Likewise, we did not find differences in the binding of IEs to the host receptor CSA in the presence of plasma samples from pregnant women with HbAA, HbAS, or HbAC [20, 21].

Increased phagocytic activity has been reported with plasma samples from children with uncomplicated malaria compared with severe malaria [41], suggesting a potential protective mechanism. Regardless of the Hb phenotype, we observed that tested plasma samples opsonize IEs and facilitate their phagocytic clearance, and older children were better at inducing it. Despite low reactivity to PfEMP1 in HbAS, not all variants associated with severe malaria were significantly lower, and seroprevalence was significant for only 2 individual PfEMP1 variants, potentially associated with uncomplicated malaria. It is probable that HbAS individuals acquire an IgG repertoire sufficient to recognize and control parasitemias before severe manifestations occur.

Some limitations identified in this study include the substantial interclonal variability of PfEMP1 antigens present in clinical isolates, which limits the detection of minor differences but is challenging to overcome. In addition, only a single domain from each studied PfEMP1 protein was included and selected, based on their relative expression levels in vitro.

In conclusion, our findings suggest that antibodies specific for PfEMP1 variants associated with severe malaria are differentially acquired in children with HbAS, probably owing to a reduced expression of PfEMP1 on the surface of the IEs. This impaired expression will likely reduce IE cytoadhesion and enable efficient splenic retention and destruction of IEs. Our findings of reduced total and sequestered parasite biomass support such a scenario. Those processes probably ameliorate severe clinical consequences of infection until adequate amounts of neutralizing and opsonizing PfEMP1-specific IgG have been acquired.

## Supplementary Data

Supplementary materials are available at *The Journal of Infectious Diseases* online (http://jid.oxfordjournals.org/). Supplementary materials consist of data provided by the author that are published to benefit the reader. The posted materials are not copyedited. The contents of all supplementary data are the sole responsibility of the authors. Questions or messages regarding errors should be addressed to the author.

## Supplementary Material

jiad438_Supplementary_Data

## Notes

***Acknowledgments.*** We thank the Ghanaian children and their families who participated in the original study; Eric Kyei-Baafour and Alex Kofi-Denso for their help with fieldwork; Anja T. R, Jensen and Michael Theisen (University of Copenhagen, Denmark) for providing the DBLβ domains and glutamate-rich recombinant protein, respectively; and Asamoah Kusi and Linda Eva Amoah (Noguchi Memorial Institute for Medical Research, University of Ghana) for providing PfCSP, PfMSP3, and Pfs230 recombinant proteins.

***Author contributions.*** A. V. O., L. H., and M. L. P. conceived and designed the study. Z. S., M. T., H. L., and M. F. O. conducted the original fieldwork. Z. S. and M. L. P. carried out the ELISA and flow cytometry experiments. A. V. O. and I. V. O. performed the bead protein array analysis. A. V. O. and M. L. P. analyzed data. M. L. P. wrote the manuscript, with inputs from all authors. All authors reviewed and approved the final version of the manuscript.

***Disclaimer.*** The funders had no role in study design, data collection and analysis, the decision to publish, or preparation of the manuscript.

***Financial support*.** This work was supported by the Independent Research Fund Denmark (grant 0134-00123B to L. H. and M. L. P.); the Danish International Development Agency (grant 17-02-KU; M. F. O. and L. H.); the Building Stronger Universities initiative grant sponsored by the Danish International Development Agency (BSUIII-UG; PhD scholarship to Z. S. and postdoctoral fellowship to H. L.); the Florida Atlantic University start-up fund (A. V. O.); and the National Institutes of Health (grant R01AI092120, A. V. O.). Funding to pay the Open Access publication charges for this article was provided by Independent Research Fund Denmark.

## References

[1] Taylor SM , Parobek CM, Fairhurst RM. Haemoglobinopathies and the clinical epidemiology of malaria: a systematic review and meta-analysis. Lancet Infect Dis 2012; 12:457–68. 22445352 10.1016/S1473-3099(12)70055-5 PMC3404513

[2] Dodoo D , Staalsoe T, Giha H, et al Antibodies to variant antigens on the surfaces of infected erythrocytes are associated with protection from malaria in Ghanaian children. Infect Immun 2001; 69:3713–8. 11349035 10.1128/IAI.69.6.3713-3718.2001 PMC98376

[3] Chan JA , Howell KB, Reiling L, et al Targets of antibodies against *Plasmodium falciparum*-infected erythrocytes in malaria immunity. J Clin Invest 2012; 122:3227–38. 22850879 10.1172/JCI62182 PMC3428085

[4] Deitsch KW , Calderwood MS, Wellems TE. Malaria: cooperative silencing elements in var genes. Nature 2001; 412:875–6. 10.1038/35091146 11528468

[5] Hviid L , Jensen AT. PfEMP1—a parasite protein family of key importance in *Plasmodium falciparum m*alaria immunity and pathogenesis. Adv Parasitol 2015; 88:51–84. 25911365 10.1016/bs.apar.2015.02.004

[6] Smith JD , Subramanian G, Gamain B, Baruch DI, Miller LH. Classification of adhesive domains in the *Plasmodium falciparum* erythrocyte membrane protein 1 family. Mol Biochem Parasitol 2000; 110:293–310. 11071284 10.1016/s0166-6851(00)00279-6

[7] Carlson J , Helmby H, Hill AV, Brewster D, Greenwood BM, Wahlgren M. Human cerebral malaria: association with erythrocyte rosetting and lack of anti-rosetting antibodies. Lancet 1990; 336:1457–60. 1979090 10.1016/0140-6736(90)93174-n

[8] Fairhurst RM , Baruch DI, Brittain NJ, et al Abnormal display of PfEMP-1 on erythrocytes carrying haemoglobin C may protect against malaria. Nature 2005; 435:1117–21. 15973412 10.1038/nature03631

[9] Cholera R , Brittain NJ, Gillrie MR, et al Impaired cytoadherence of *Plasmodium falciparum*-infected erythrocytes containing sickle hemoglobin. Proc Natl Acad Sci U S A 2008; 105:991–6. 18192399 10.1073/pnas.0711401105 PMC2242681

[10] Sanchez CP , Karathanasis C, Sanchez R, et al Single-molecule imaging and quantification of the immune-variant adhesin VAR2CSA on knobs of plasmodium falciparum-infected erythrocytes. Commun Biol 2019; 2:172. 31098405 10.1038/s42003-019-0429-z PMC6506540

[11] Seidu Z , Ofori MF, Hviid L, Lopez-Perez M. Impact of sickle cell trait hemoglobin in *Plasmodium falciparum*-infected erythrocytes. BioRxiv [Preprint: not peer reviewed]. 28 July 2023. Available from: https://www.biorxiv.org/content/10.1101/2023.07.28.551025v1.

[12] Lansche C , Dasanna AK, Quadt K, et al The sickle cell trait affects contact dynamics and endothelial cell activation in *Plasmodium falciparum*-infected erythrocytes. Commun Biol 2018; 1:211. 30534603 10.1038/s42003-018-0223-3 PMC6269544

[13] Loiseau C , Traore B, Ongoiba A, et al Memory CD8^+^ T cell compartment associated with delayed onset of *Plasmodium falciparum* infection and better parasite control in sickle-cell trait children. Clin Transl Immunology 2021; 10:e1265. 33763229 10.1002/cti2.1265 PMC7979311

[14] Loiseau C , Doumbo OK, Traore B, et al A novel population of memory-activated natural killer cells associated with low parasitaemia in *Plasmodium falciparum*-exposed sickle-cell trait children. Clin Transl Immunology 2020; 9:e1125. 32257211 10.1002/cti2.1125 PMC7114700

[15] Lopera-Mesa TM , Doumbia S, Konate D, et al Effect of red blood cell variants on childhood malaria in Mali: a prospective cohort study. Lancet Haematol 2015; 2:e140–9. 26687956 10.1016/S2352-3026(15)00043-5 PMC4418020

[16] Allen SJ , Bennett S, Riley EM, et al Morbidity from malaria and immune responses to defined *Plasmodium falciparum* antigens in children with sickle cell trait in the Gambia. Trans R Soc Trop Med Hyg 1992; 86:494–8. 1475814 10.1016/0035-9203(92)90083-o

[17] Le Hesran JY , Personne I, Personne P, et al Longitudinal study of *Plasmodium falciparum* infection and immune responses in infants with or without the sickle cell trait. Int J Epidemiol 1999; 28:793–8. 10480713 10.1093/ije/28.4.793

[18] Verra F , Simpore J, Warimwe GM, et al Haemoglobin C and S role in acquired immunity against *Plasmodium falciparum* malaria. PLoS One 2007; 2:e978. 17912355 10.1371/journal.pone.0000978 PMC1991593

[19] Tan X , Traore B, Kayentao K, et al Hemoglobin S and C heterozygosity enhances neither the magnitude nor breadth of antibody responses to a diverse array of *Plasmodium falciparum* antigens. J Infect Dis 2011; 204:1750–61. 21998476 10.1093/infdis/jir638 PMC3203232

[20] Lopez-Perez M , Viwami F, Seidu Z, et al PfEMP1-specific immunoglobulin G reactivity among Beninese pregnant women with sickle cell trait. Open Forum Infect Dis 2021; 8:ofab527. 34909438 10.1093/ofid/ofab527 PMC8664683

[21] Lopez-Perez M , Viwami F, Doritchamou J, Ndam NT, Hviid L. Natural acquired immunity to malaria antigens among pregnant women with hemoglobin C trait. Am J Trop Med Hyg 2022; 106:853–6. 35026728 10.4269/ajtmh.21-1039 PMC8922521

[22] Seidu Z , Lamptey H, Lopez-Perez M, et al *Plasmodium falciparum* infection and naturally acquired immunity to malaria antigens among Ghanaian children in Northern Ghana. Parasite Epidemiol Control 2023; 22:e00317. 37501921 10.1016/j.parepi.2023.e00317 PMC10369471

[23] Tetteh M , Addai-Mensah O, Siedu Z, et al Acute phase responses vary between children of HbAS and HbAA genotypes during *Plasmodium falciparum* infection. J Inflamm Res 2021; 14:1415–26. 33889007 10.2147/JIR.S301465 PMC8055362

[24] Lamptey H , Seidu Z, Lopez-Perez M, et al Impact of haemoglobinopathies on asymptomatic *Plasmodium falciparum* infection and naturally acquired immunity among children in Northern Ghana. Front Hematol 2023; 2:1150134. 10.1016/j.parepi.2023.e00317 PMC10369471 37501921

[25] Hendriksen IC , Mwanga-Amumpaire J, von Seidlein L, et al Diagnosing severe falciparum malaria in parasitaemic African children: a prospective evaluation of plasma PfHRP2 measurement. PLoS Med 2012; 9:e1001297. 22927801 10.1371/journal.pmed.1001297 PMC3424256

[26] Stevenson L , Laursen E, Cowan GJ, et al α2-Macroglobulin can crosslink multiple *Plasmodium falciparum* erythrocyte membrane protein 1 (PfEMP1) molecules and may facilitate adhesion of parasitized erythrocytes. PLoS Pathog 2015; 11:e1005022. 26134405 10.1371/journal.ppat.1005022 PMC4489720

[27] Olsen RW , Ecklu-Mensah G, Bengtsson A, et al Acquisition of IgG to ICAM-1-binding DBLβ domains in the *Plasmodium falciparum* erythrocyte membrane protein 1 antigen family varies between groups A, B, and C. Infect Immun 2019; 87:e00224–19. 31308082 10.1128/IAI.00224-19 PMC6759304

[28] Theisen M , Vuust J, Gottschau A, Jepsen S, Hogh B. Antigenicity and immunogenicity of recombinant glutamate-rich protein of *Plasmodium falciparum* expressed in *Escherichia coli*. Clin Diagn Lab Immunol 1995; 2:30–4. 7719909 10.1128/cdli.2.1.30-34.1995 PMC170096

[29] Amoah LE , Acquah FK, Ayanful-Torgby R, et al Dynamics of anti-MSP3 and Pfs230 antibody responses and multiplicity of infection in asymptomatic children from southern Ghana. Parasit Vectors 2018; 11:13. 29304870 10.1186/s13071-017-2607-5 PMC5755320

[30] Acquah FK , Obboh EK, Asare K, et al Antibody responses to two new *Lactococcus lactis*-produced recombinant Pfs48/45 and Pfs230 proteins increase with age in malaria patients living in the Central Region of Ghana. Malar J 2017; 16:306. 28764709 10.1186/s12936-017-1955-0 PMC5540549

[31] Oleinikov AV , Amos E, Frye IT, et al High throughput functional assays of the variant antigen PfEMP1 reveal a single domain in the 3D7 *Plasmodium falciparum* genome that binds ICAM1 with high affinity and is targeted by naturally acquired neutralizing antibodies. PLoS Pathog 2009; 5:e1000386. 19381252 10.1371/journal.ppat.1000386 PMC2663049

[32] Oleinikov AV , Voronkova VV, Frye IT, et al A plasma survey using 38 PfEMP1 domains reveals frequent recognition of the *Plasmodium falciparum* antigen VAR2CSA among young Tanzanian children. PLoS One 2012; 7:e31011. 22295123 10.1371/journal.pone.0031011 PMC3266279

[33] Lopez-Perez M , Seidu Z. Establishing and maintaining in vitro cultures of asexual blood stages of *Plasmodium falciparum*. Methods Mol Biol 2022; 2470:37–49. 35881337 10.1007/978-1-0716-2189-9_5

[34] Lopez-Perez M , Olsen RW. Immunomagnetic selection of *Plasmodium falciparum*-infected erythrocytes expressing particular PfEMP1 variants. Methods Mol Biol 2022; 2470:69–78. 35881339 10.1007/978-1-0716-2189-9_7

[35] Hedberg P , Sirel M, Moll K, et al Red blood cell blood group A antigen level affects the ability of heparin and PfEMP1 antibodies to disrupt *Plasmodium falciparum* rosettes. Malar J 2021; 20:441. 34794445 10.1186/s12936-021-03975-w PMC8600353

[36] Teo A , Hasang W, Boeuf P, Rogerson S. A robust phagocytosis assay to evaluate the opsonic activity of antibodies against *Plasmodium falciparum*-infected erythrocytes. Methods Mol Biol 2015; 1325:145–52. 26450386 10.1007/978-1-4939-2815-6_12

[37] Stevenson L , Huda P, Jeppesen A, et al Investigating the function of Fc-specific binding of IgM to *Plasmodium falciparum* erythrocyte membrane protein 1 mediating erythrocyte rosetting. Cell Microbiol 2015; 17:819–31. 25482886 10.1111/cmi.12403 PMC4737123

[38] Lennartz F , Adams Y, Bengtsson A, et al Structure-guided identification of a family of dual receptor-binding PfEMP1 that is associated with cerebral malaria. Cell Host Microbe 2017; 21:403–14. 28279348 10.1016/j.chom.2017.02.009 PMC5374107

[39] Chesnokov O , Merritt J, Tcherniuk SO, Milman N, Oleinikov AV. *Plasmodium falciparum* infected erythrocytes can bind to host receptors integrins αVβ3 and αVβ6 through DBLδ1_D4 domain of PFL2665c PfEMP1 protein. Sci Rep 2018; 8:17871. 30552383 10.1038/s41598-018-36071-2 PMC6294747

[40] Chan JA , Boyle MJ, Moore KA, et al Antibody targets on the surface of *Plasmodium falciparum*-infected erythrocytes that are associated with immunity to severe malaria in young children. J Infect Dis 2019; 219:819–28. 30365004 10.1093/infdis/jiy580 PMC6376912

[41] Suurbaar J , Moussiliou A, Tahar R, et al ICAM-1-binding *Plasmodium falciparum* erythrocyte membrane protein 1 variants elicits opsonic-phagocytosis IgG responses in Beninese children. Sci Rep 2022; 12:12994. 35906450 10.1038/s41598-022-16305-0 PMC9338288

[42] Andolina C , Ramjith J, Rek J, et al *Plasmodium falciparum* gametocyte carriage in longitudinally monitored incident infections is associated with duration of infection and human host factors. Sci Rep 2023; 13:7072. 37127688 10.1038/s41598-023-33657-3 PMC10150352

[43] Gupta S , Snow RW, Donnelly CA, Marsh K, Newbold C. Immunity to non-cerebral severe malaria is acquired after one or two infections. Nat Med 1999; 5:340–3. 10086393 10.1038/6560

[44] Goncalves BP , Huang CY, Morrison R, et al Parasite burden and severity of malaria in Tanzanian children. N Engl J Med 2014; 370:1799–808. 24806160 10.1056/NEJMoa1303944 PMC4091983

[45] Cham GK , Turner L, Kurtis JD, et al Hierarchical, domain type-specific acquisition of antibodies to *Plasmodium falciparum* erythrocyte membrane protein 1 in Tanzanian children. Infect Immun 2010; 78:4653–9. 20823214 10.1128/IAI.00593-10 PMC2976311

[46] Obeng-Adjei N , Larremore DB, Turner L, et al Longitudinal analysis of naturally acquired PfEMP1 CIDR domain variant antibodies identifies associations with malaria protection. JCI Insight 2020; 5:e137262. 32427581 10.1172/jci.insight.137262 PMC7406271

[47] Duffy MF , Noviyanti R, Tsuboi T, et al Differences in PfEMP1s recognized by antibodies from patients with uncomplicated or severe malaria. Malar J 2016; 15:258. 27149991 10.1186/s12936-016-1296-4 PMC4858840

[48] Doumbo OK , Thera MA, Kone AK, et al High levels of *Plasmodium falciparum* rosetting in all clinical forms of severe malaria in African children. Am J Trop Med Hyg 2009; 81:987–93. 19996426 10.4269/ajtmh.2009.09-0406 PMC2877664

