# Supplementary material for: Profiling the Plasmodium falciparum Erythrocyte Membrane Protein 1–Specific Immununoglobulin G Response Among Ghanaian Children With Hemoglobin S and C
Source: J Infect Dis. 2023 Oct 6;229(1):203–13. doi: 10.1093/infdis/jiad438 (PMC10786258; doi:10.1093/infdis/jiad438)
Supplement: jiad438_Supplementary_Data [file jiad438_supplementary_data.docx]

**Supplementary material**

**Profiling the PfEMP1-specific IgG response among Ghanaian children with hemoglobin S and C**

Andrew V. Oleinikov^1*^, Zakaria Seidu ^2, 3, 4*‡^, Irina V. Oleinikov^1^, Mary Tetteh^5‡^, Helena Lamptey^3^, Michael F. Ofori^3^, Lars Hviid^2,6^, Mary Lopez-Perez^2#^

^[[1]](#footnote-1)^Charles E Schmidt College of Medicine, Florida Atlantic University, Boca Raton, FL, USA; ^2^Centre for Medical Parasitology, Department of Immunology and Microbiology, Faculty of Health and Medical Sciences, University of Copenhagen, Copenhagen, Denmark; ^3^Department of Immunology, Noguchi Memorial Institute for Medical Research, College of Health Sciences, University of Ghana, Accra, Ghana; ^4^West Africa Centre for Cell Biology of Infectious Pathogens, Department of Biochemistry, Cell and Molecular Biology, University of Ghana, Accra Ghana; ^5^Department of Medical Diagnostics, Faculty of Allied Health Sciences, Kwame Nkrumah University of Science and Technology, Kumasi, Ghana; ^6^Centre for Medical Parasitology, Department of Infectious Diseases, Rigshospitalet, Copenhagen, Denmark.

# Supplementary Figures

## ***Supplementary Figure 1. Antibody response to native PfEMP1.***

Specific IgG levels to HB3VAR06, IT4VAR09, and IT4VAR60 expressed on the surface of IEs and measured by flow cytometry in HbAA, HbAS, and HbAC samples. Medians and p values using the Kruskal-Wallis test followed by Dunn's multiple comparisons test are shown. Values are expressed as mean fluorescence intensity (nMFI).


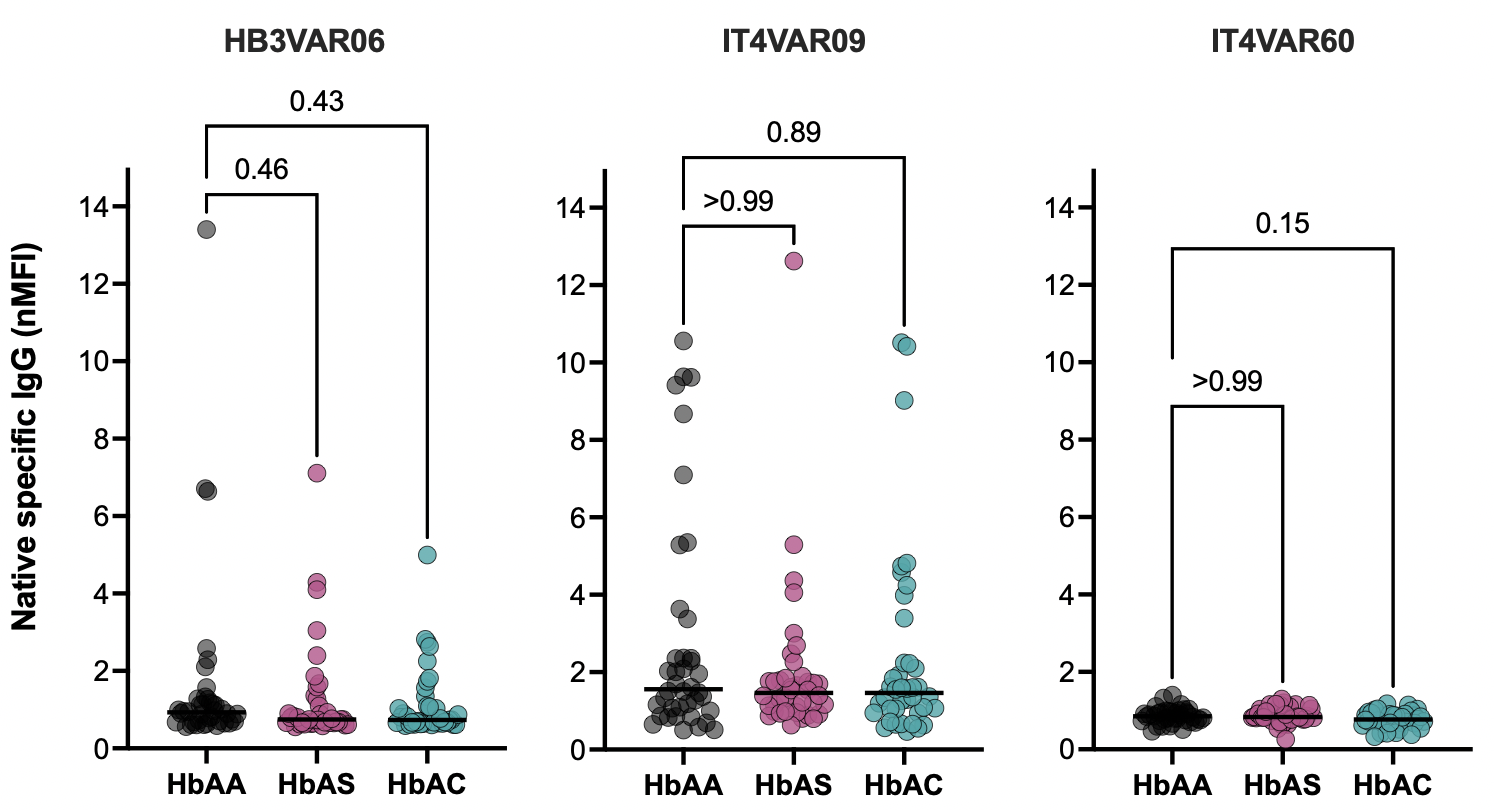


## ***Supplementary Figure 2. Seroprevalence of IgG response to PfEMP1 domains.***

Heatmap showing seroprevalence to each of the 40 PfEMP1 proteins. Antigens were ranked according to the seroprevalence (color bar) and grouped according to the Hb genotype. Black arrow: PfEMP1 protein containing an EPCR-binding domain, orange arrow: PfEMP1 protein containing an ICAM-1-binding domain. *p < 0.05.


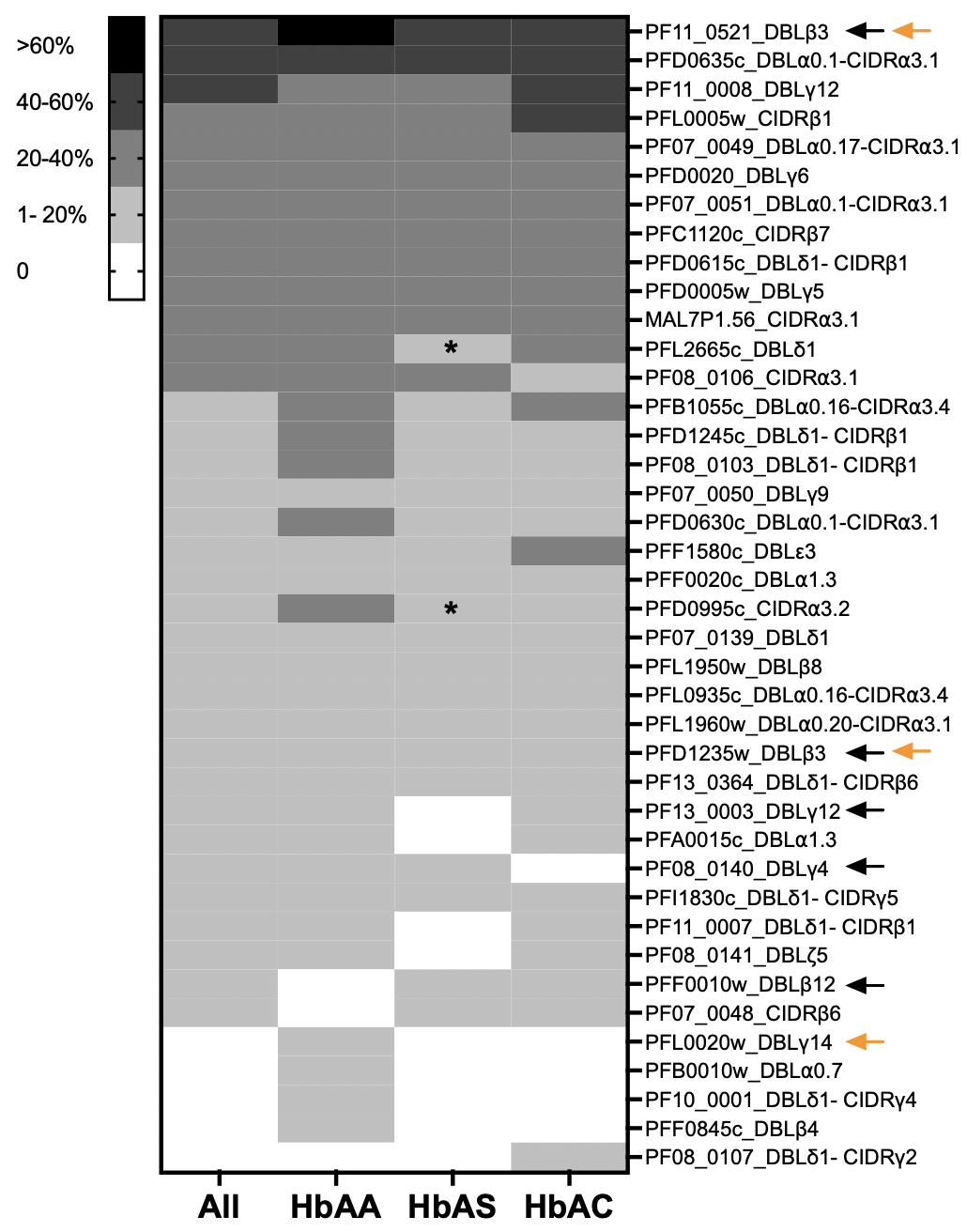


## ***Supplementary Figure 3. Age-dependence of PfEMP1-specific IgG response.***

Simple linear regression for age plotted against the breadth of IgG reactivity to PfEMP1 proteins grouped according to *var* gene. Spearman’s rank correlation and p values for 120 children (40 for each blood type, but 37 HbAS and 39 HbAC tested for groups A and B/A) are shown.

*
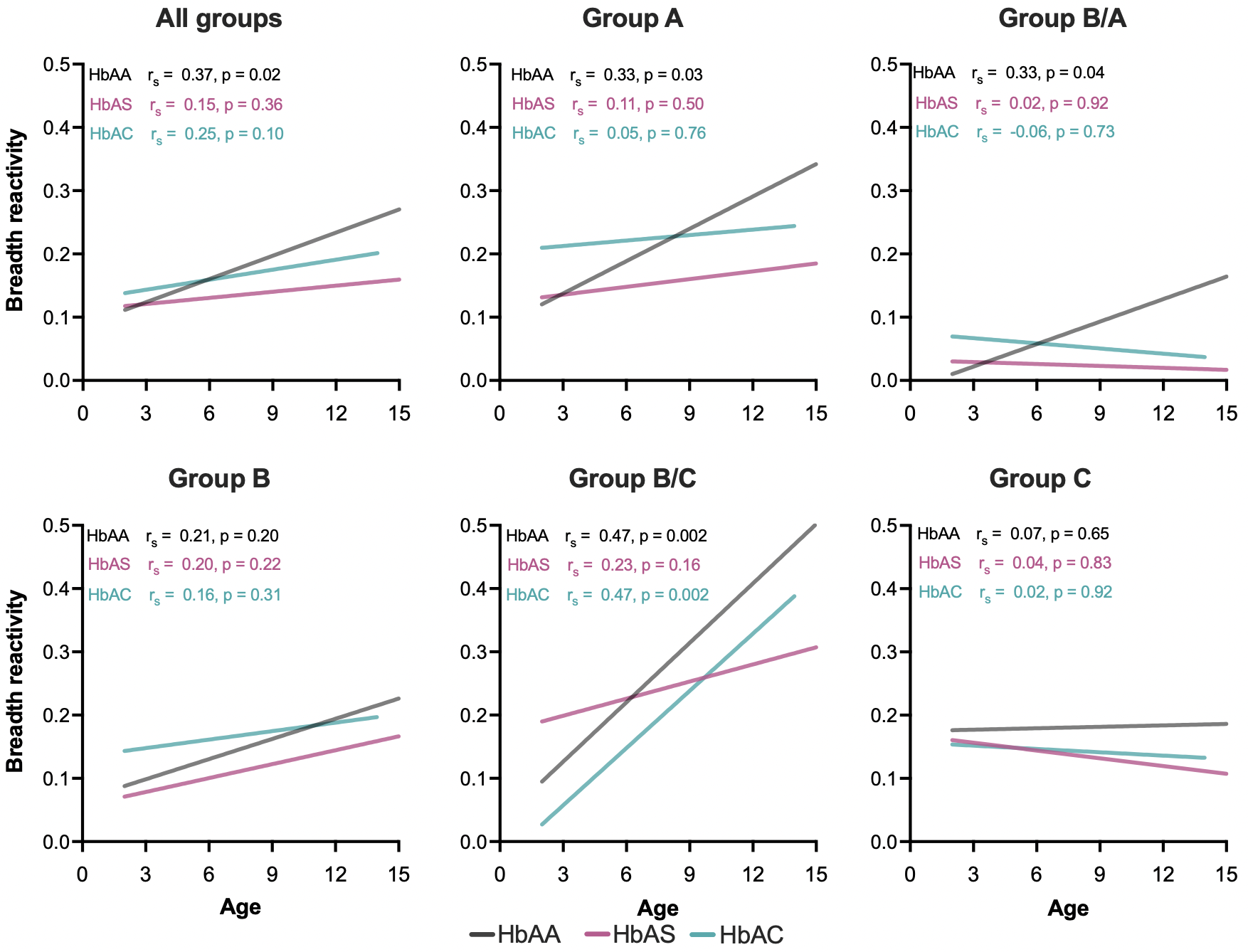
*

## ***Supplementary Figure 4. Rosette disruption by plasma samples***

Rosette disruption by plasma from HbAA (n = 27), HbAS (n = 31), and HbAC (n = 24) children was measured as the rosette size relative to the absence of specific PfEMP1 antibodies (control for maximum rosetting; dotted line). P values using the Kruskal-Wallis test followed by Dunn's multiple comparisons test and medians are shown.


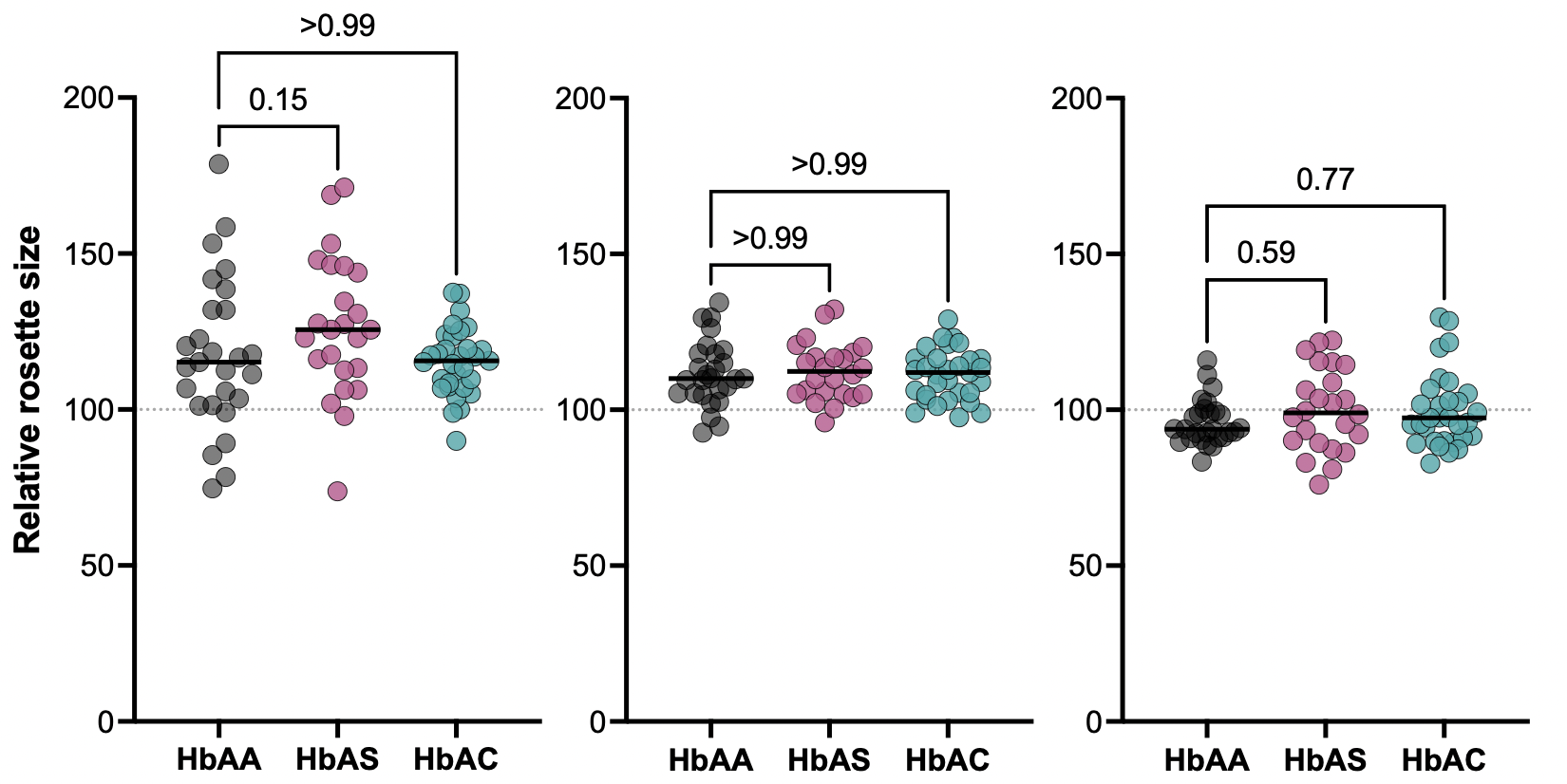


## ***Supplementary Figure 5. Age-dependence of rosetting rate and PfEMP1-specific IgG dependent phagocytosis.***

(**A**) Rosetting rate in the presence of plasma from 2-5y (n = 23), 6-9y (n = 23), and 10-15y (n = 35) children and relative to the absence of specific PfEMP1 antibodies (control for maximum rosetting; dotted line). (**B**) Phagocytes of IEs by plasma from the same children relative to the positive control (control for maximum phagocytosis; dotted line). P values using the Kruskal-Wallis test followed by Dunn's multiple comparisons test and medians are shown.

**
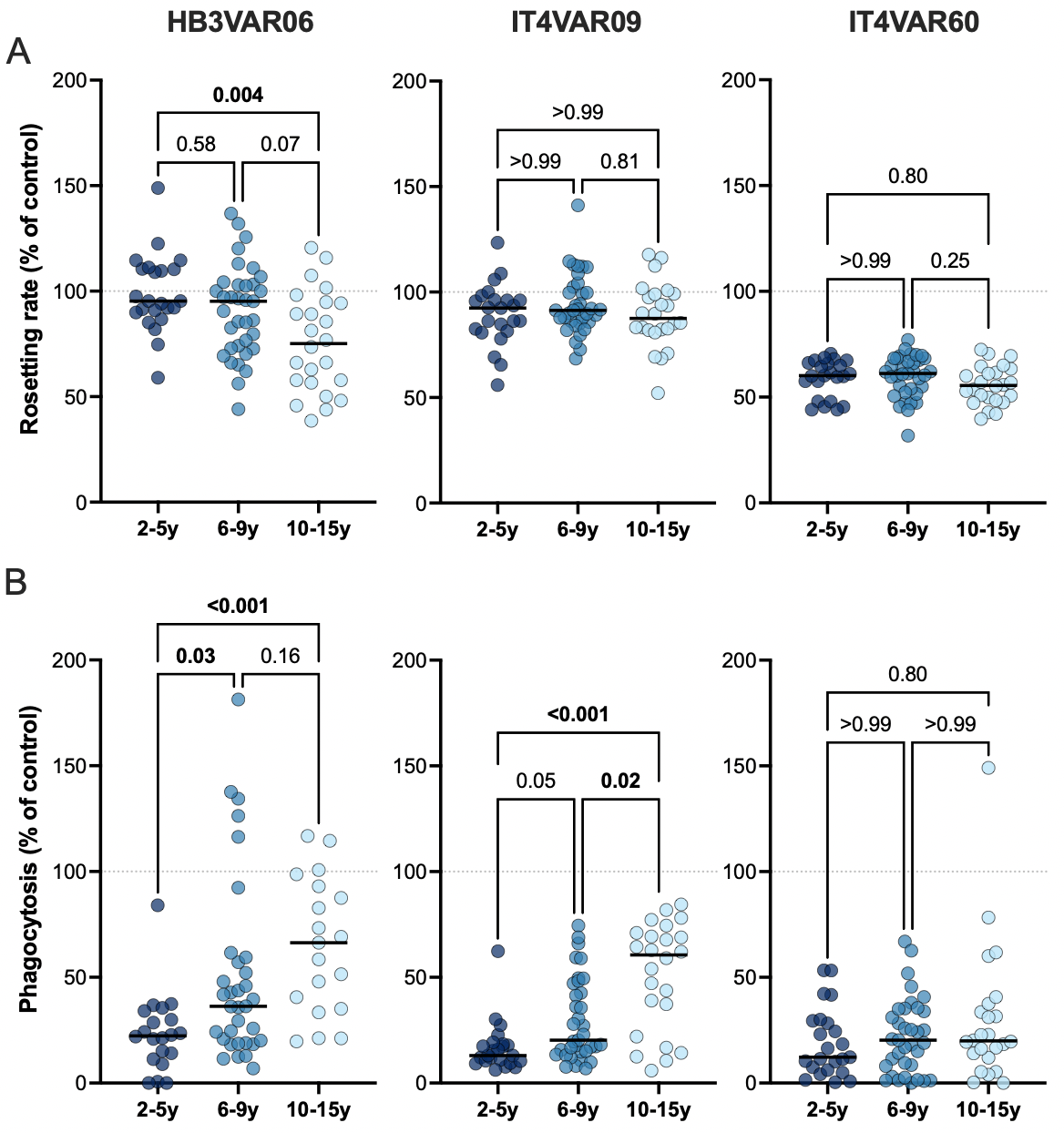
**

# Supplementary Tables

# *Supplementary Table 1. List of PfEMP1 domains used in the beads assay*

| **Protein ID** | **Domain(s) tested** | **Domain class** | **CIDR domain class*^a^*** | **PfEMP1 group** | **Binding phenotype*^b^*** |
| --- | --- | --- | --- | --- | --- |
| PFD1235w | DBLβ3 | DBLβ | CIDR⍺1 | A | EPCR/ICAM1 |
| PF11_0521 | DBLβ3 | DBLβ | CIDR⍺1 | A | EPCR/ICAM1 |
| PF13_0003 | DBL𝛾12 | DBL𝛾 | CIDRδ | A | EPCR/Rosetting |
| PF08_0141 | DBL𝜁5 | DBL𝜁 | CIDRδ | A | Spectrin/IgM |
| PF11_0008 | DBL𝛾12 | DBL𝛾 | CIDR𝛾 | A | PECAM1/IgM |
| PFD0020 | DBL𝛾6 | DBL𝛾 | CIDR⍺1 | A | gC1qR |
| PFA0015c | DBL⍺1.3 | DBL⍺ | NA | A | Unknown |
| PFF0020c | DBL⍺1.3 | DBL⍺ | NA | A | Unknown |
| PF08_0140 | DBL𝛾4 | DBL𝛾 | CIDR⍺1 | B/A | EPCR |
| PFF0010w | DBLβ12 | DBLβ | CIDR⍺1 | B/A | EPCR/IgM |
| PFL0020w | DBL𝛾14 | DBL𝛾 | CIDR⍺2-6 | B/A | IgM/ICAM1 |
| PFF1580c | DBLε3 | DBLε | CIDR⍺2-6 | B/A | IgM |
| PF11_0007 | DBLδ1- CIDRβ1 | DBLδ- CIDRβ | CIDR⍺2-6 | B | IgM |
| PFC1120c | CIDRβ7 | CIDRβ | CIDR⍺2-6 | B | Unknown |
| PFD0005w | DBL𝛾5 | DBL𝛾 | CIDR⍺2-6 | B | CD36 |
| PF13_0364 | DBLδ1- CIDRβ6 | DBLδ- CIDRβ | CIDR⍺2-6 | B | CD36 |
| PF07_0139 | DBLδ1 | DBLδ | CIDR⍺2-6 | B | IgM |
| PFB1055c | DBL⍺0.16-CIDR⍺3.4 | DBL⍺-CIDR⍺ | CIDR⍺2-6 | B | CD36 |
| PFL0005w | CIDRβ1 | CIDRβ | CIDR⍺2-6 | B | CD36 |
| PFB0010w | DBL⍺0.7 | DBL⍺ | CIDR⍺2-6 | B | CD36/αVβ3/6 integrin |
| PFD1245c | DBLδ1- CIDRβ1 | DBLδ- CIDRβ | CIDR⍺2-6 | B | CD36 |
| PFL0935c | DBL⍺0.16-CIDR⍺3.4 | DBL⍺-CIDR⍺ | CIDR⍺2-6 | B | CD36 |
| PFL2665c | DBLδ1 | DBLδ | CIDR⍺2-6 | B | CD36/αVβ3/6 integrin |
| PFI1830c | DBLδ1- CIDR𝛾5 | DBLδ- CIDR𝛾 | CIDR⍺2-6 | B | CD36 |
| PF10_0001 | DBLδ1- CIDR𝛾4 | DBLδ- CIDR𝛾 | CIDR⍺2-6 | B | CD36 |
| PF08_0103 | DBLδ1- CIDRβ1 | DBLδ- CIDRβ | CIDR⍺2-6 | B/C | CD36/IgM |
| PFD0635c | DBL⍺0.1-CIDR⍺3.1 | DBL⍺-CIDR⍺ | CIDR⍺2-6 | B/C | Unknown |
| PF08_0106 | CIDR⍺3.1 | CIDR⍺ | CIDR⍺2-6 | B/C | CD36 |
| PFL1950w | DBLβ8 | DBLβ | CIDR⍺2-6 | B/C | Unknown |
| PF07_0050 | DBL𝛾9 | DBL𝛾 | CIDR⍺2-6 | B/C | Unknown |
| MAL7P1.56 | CIDR⍺3.1 | CIDR⍺ | CIDR⍺2-6 | C | Unknown |
| PF08_0107 | DBLδ1- CIDR𝛾2 | DBLδ- CIDR𝛾 | CIDR⍺2-6 | C | Unknown |
| PFD0995c | CIDR⍺3.2 | CIDR⍺ | CIDR⍺2-6 | C | CD36 |
| PF07_0049 | DBL⍺0.17-CIDR⍺3.1 | DBL⍺-CIDR⍺ | CIDR⍺2-6 | C | Unknown |
| PFD0630c | DBL⍺0.1-CIDR⍺3.1 | DBL⍺-CIDR⍺ | CIDR⍺2-6 | C | Unknown |
| PFD0615c | DBLδ1- CIDRβ1 | DBLδ- CIDRβ | CIDR⍺2-6 | C | Unknown |
| PF07_0051 | DBL⍺0.1-CIDR⍺3.1 | DBL⍺-CIDR⍺ | CIDR⍺2-6 | C | IgM |
| PF07_0048 | CIDRβ6 | CIDRβ | CIDR⍺2-6 | C | IgM |
| PFL1960w | DBL⍺0.20-CIDR⍺3.1 | DBL⍺-CIDR⍺ | CIDR⍺2-6 | C | CD36 |
| PFF0845c | DBLβ4 | DBLβ | CIDR⍺2-6 | C | Unknown |
|  |  |  |  |  |  |
| **Protein ID*^c^*** | **Domain(s) tested** | **Domain class** | **CIDR domain class*^a^*** | **PfEMP1 group** | **Binding phenotype*^b^*** |
| PF08_0142 | DBLδ1- CIDRβ1 | DBLδ- CIDRβ | CIDR⍺2-6 | B | CD36 |
| PFE0005w | DBLδ1- CIDRβ1 | DBLδ- CIDRβ | CIDR⍺2-6 | B | IgM |
| PFI0005w | DBLδ1- CIDR𝛾12 | DBLδ- CIDR𝛾 | CIDR⍺2-6 | B | CD36 |
| PFA0765c | DBLδ1 | DBLδ | CIDR⍺2-6 | B | CD36/αVβ3/6 integrin |
| PFL1955w | DBLδ1 | DBLδ | CIDR⍺2-6 | B/C | CD36/αVβ3/6 integrin |
| MAL7P1.55 | DBL⍺0.9-CIDR⍺2.4 | DBL⍺-CIDR⍺ | CIDR⍺2-6 | B/C | Unknown |

*^a^*CIDR domain present in the protein but not tested in this study; *^b^*Binding phenotype for the PfEMP1 protein; *^c^*included in the beads array but not in the analyses since they were not recognized by plasma from children.

# *Supplementary Table 2. Demographic characteristics in the first cohort: children with P. falciparum infection from Northern Ghana*

| **Parameter** | **HbAA (n = 96)** |  | **HbAS (n = 12)** |  | **HbAC (n = 27)** |  | **Total (n = 135)** |  | **p value*^a^*** |
| --- | --- | --- | --- | --- | --- | --- | --- | --- | --- |
| Female | 23 (24%) |  | 5 (42%) |  | 10 (44%) |  | 38 (30%) |  | 0.08 |
| Submicroscopic parasitaemia*^b^* | 48 (49%) |  | 3 (25%) |  | 14 (52%) |  | 65 (47%) |  | 0.26 |
| Age (years) | 8 [6-11] |  | 5 [4-9] |  | 8 [5-10] |  | 8 [6-10] |  | 0.22 |
| Hb (g/dL) | 10.8 [10.1‑11.6] |  | 10.7 [9.6‑11.5] |  | 10.5 [9.8‑11.3] |  | 10.6 [10.0‑11.6] |  | 0.34 |

*^a^*p value using Chi-square test for qualitative variables and Kruskal‑Wallis test for quantitative variables. *^b^*Positive PCR but negative for microscopy. Interquartile ranges are presented in brackets.

# *Supplementary Table 3. Demographic characteristics in the second cohort: children with uncomplicated malaria from Begoro*

| **Parameter** | **HbAA (n = 45)** |  | **HbAS (n = 13)** |  | **Total (n = 58)** |  | **p value*^a^*** |
| --- | --- | --- | --- | --- | --- | --- | --- |
| Female | 18 (40%) |  | 9 (69%) |  | 27 (47%) |  | 0.06 |
| Age (years) | 7 [4-10] |  | 5 [3-11] |  | 6 [4-10] |  | 0.44 |
| Hb (g/dL) | 10.3 [8.9-11.6] |  | 10.2 [1.0-11.6] |  | 10.3 [9.0-11.5] |  | 0.92 |

*^a^*p value using Chi-square test for qualitative variables and Mann-Whitney test for quantitative variables. Interquartile ranges are presented in brackets.

1. ***^#^Correspondence.*** Mary Lopez-Perez, Panum Institute 07-11-38, Blegdamsvej 3B, 2200 Copenhagen N, Denmark ([mlopez@sund.ku.dk](mailto:mlopez@sund.ku.dk)).

   *Those authors contribute equally to this work.

   ^‡^***Current affiliation*.** Zakaria Seidu, Department of Biochemistry and Molecular Biology, Faculty of Biosciences, University for Development Studies, Nyankpala, Ghana.

   Mary Tetteh, Ghana Health Service, Ashaiman Municipal Hospital, Accra. [↑](#footnote-ref-1)
